# Dexmedetomidine-Fentanyl versus Midazolam-Fentanyl in Pain Management of Distal Radius Fractures Reduction; a Randomized Clinical Trial

**Published:** 2018-01-20

**Authors:** Ali Arhami Dolatabadi, Elham Memary, Majid Shojaee, Hossein Kamalifard

**Affiliations:** 1 Emergency Department, Imam Hossein Hospital, Shahid Beheshti University Of Medical Sciences, Tehran, Iran.; 2 Anesthesiology Department, Imam Hossein Hospital, Faculty of Medicine, Shahid Beheshti University of Medical Sciences, Tehran, Iran.

**Keywords:** Conscious sedation, analgesia, dexmedetomidine, midazolam, closed fracture reduction, clinical trial

## Introduction

### Introduction:

Currently, using various combinations of sedative and analgesic agents has received attention for induction of sedation and analgesia due to their synergy in controlling pain and anxiety. The present study was designed with the aim of comparing dexmedetomidine-fentanyl combination with midazolam-fentanyl in this regard.

### Methods:

 In this randomized clinical trial, patients diagnosed with distal radius fracture who had visited the emergency department (ED) were allocated to either the group receiving the combination of fentanyl-midazolam or the one receiving dexmedetomidine-fentanyl for procedural sedation and analgesia (PSA) and were compared regarding analgesic characteristics, time to recovery and side effects.

### Results:

80 patients with the mean age of 42.08 ± 12.17 (18 - 60) years were randomly allocated to 2 groups of 40 (83.80% male). The 2 groups did not have a significant difference regarding baseline characteristics as well as pain severity. Mean pain score at the time of procedure was 3.47 ± 1.37 in dexmedetomidine and 2.85 ± 1.05 in midazolam group (p = 0.025). In addition, time to recovery in dexmedetomidine and midazolam groups was 6.60 ± 1.86 minutes and 12.70 ± 1.70 minutes, respectively (p < 0.001). Out of the 9 patients who experienced treatment failure, 8 (88.90%) patients were in dexmedetomidine group and 1 (11.10%) was in midazolam group (p = 0.029). Absolute risk increase rate of treatment failure in case of using dexmedetomidine instead of midazolam was 17.50% (95%CI: 4.19 – 30.81) and number needed to harm was 6.00 (95% CI: 3.20 – 23.80).

### Conclusion:

Although the combination of dexmedetomidine-fentanyl had a shorter time to recovery compared to midazolam-fentanyl for induction of sedation and analgesia, the treatment failure rate in case of using dexmedetomidine with 1 µg/kg increased 17.5% and about 1 out of each 6 patients needed a rescue dose. 

## Introduction:

Distal radius fracture is among the common orthopedic problems and preserving the function of the joint by reduction, anatomic correction and fixating under sedation and analgesia are the priorities of treating these patients in emergency department (ED) (1). For reducing pain at the time of reduction, various methods exist including nerve block (2), hematoma block (3), and induction of sedation and analgesia using various drug compounds available to the physicians handling these patients (4, 5).

Fewer side effects on the respiratory system and hemodynamic status are among the characteristics desired by emergency physicians when selecting a drug for use in induction of sedation and analgesia. Currently, using various combinations of sedative and analgesic agents has received attention in this regard due to their synergy in controlling pain and anxiety. For this purpose, the combination of midazolam and fentanyl, as a powerful and short acting synthetic opiate and a fast acting benzodiazepine with a short half-life, respectively, has been very desirable and frequently used among emergency medicine specialists (6, 7).

On the other hand, dexmedetomidine as a central alpha-2 agonist that has received the official approval of American food and drug administration (FDA) in 1999 and has sympatholytic, anti-anxiety, pain relief, and analgesic effects has been considered for induction of conscious sedation (8).

Findings of various studies on comparison of the sedative effects of dexmedetomidine and midazolam is indicative of their difference regarding time of recovery and side effects on the respiratory system and hemodynamics (9, 10). Although a study by Vazquez et al. indicated that the time to recovery of midazolam was twice the time to recovery of dexmedetomidine (11), another study by Zeyneloglu et al. showed completely reversed results (12). It seems that dexmedetomidine will have a more successful performance compared to midazolam in cases that sedation is required for airway procedures such as bronchoscopy (13). Therefore, the present study was designed and performed with the aim of comparing dexmedetomidine and fentanyl combination with midazolam and fentanyl combination in induction of sedation and analgesia for reduction of distal radius fracture in the ED.

## Methods:

***Study design and setting***

In the present single blind randomized clinical trial, patients diagnosed with distal radius fracture who had visited the ED of Imam Hossein and Hafte Tir Hospitals, Tehran, Iran, were studied. The patients were allocated to either the group receiving the combination of fentanyl and midazolam (midazolam group) or the one receiving dexmedetomidine and fentanyl (dexmedetomidine group) for induction of sedation and analgesia for reducing the fracture and the characteristics of the 2 combinations were compared. This study was approved by the ethics committee of faculty of medicine, Shahid Beheshti University of Medical Sciences and was registered on the Iranian registry of clinical trials (IRCT) under the number IRCT20160401027165N1. The researchers adhered to ethical principles and confidentiality of patient data throughout the study. Informed consent was obtained from the patients for participating in the study.

***Participants***

 All the patients with distal radius fractures who had presented in the working shifts of the senior resident in charge of the study (including morning and night shifts in both weekdays and holidays) and aged between 18 and 60 years were included in the study without any sex limitations and using non-probability consecutive sampling. Patients who had a history of using antihypertensive or antihistamine medications, patients with head trauma and loss of consciousness, severe chest trauma, cervical vertebra trauma with unstable fracture, mental retardation, those who could not verbally communicate, hemodynamically unstable patients, those with a history of allergic reaction to drugs, addicts and those who had a history of drug abuse, pregnant women, and those with a history of cardiac disease (cardiac block and bradycardia) were excluded from the study.

***Intervention***

After selecting the patients meeting the inclusion criteria, the participants were randomly allocated to one of the study groups. After establishment of proper peripheral vein, cardiac monitoring, pulse oximetry, blood pressure monitoring and preparing complete equipment for cardiopulmonary resuscitation on the patients’ bedside, attempts were made to induce sedation and analgesia at the level of conscious sedation. The patient and the person who was responsible for statistical analysis were blind to the type of drug used. A trained nurse was in charge of preparing the 2 drug compounds used in separate syringes looking the same. In addition, a senior emergency medicine resident was in charge of the study and data gathering under the supervision of an emergency medicine specialist. We cannot be sure of the blinding of the person gathering data to the type of treatment received, due to the difference in the method of injecting midazolam and dexmedetomidine and not performing double dummy blinding.

In this study, dose of dexmedetomidine (Huspiria of USA, Behestan Phamaceutical CO, Iran) was considered 1µg/kg and was injected during 10 minutes in 100cc normal saline. In addition, midazolam (Darupakhsh Co, Iran) with dose of 0.01 mg/kg was administered via slow and titrated intravenous injection. Fentanyl (Abu Ravihan Co, Iran) was prescribed with 3 µg/kg body weight dose for both groups and was administered via slow intravenous injection.

Pain severity of the patients was measured and recorded using visual analogue scale (VAS) once before administration of the drugs and once before performing the procedure (10-15 minutes after drug administration). A score of 10 was considered the worst pain score and 0 was the lowest score. At least 3 points decrease in pain severity 10-15 minutes after receiving the drug was considered as treatment success and not decreasing as much was considered treatment failure.

In case the pain did not decrease in a maximum of 15 minutes after drug injection, another injection was done using fentanyl with 3 µg/kg body weight dose but these patients were not excluded from the final analysis.

***Data gathering***

To gather data, a designed checklist consisting of age, sex, pain severity before intervention and 15 minutes after receiving the drug, duration of procedure, time to recovery, and probable side effects (apnea, nausea and vomiting, hypotension, and bradycardia) was used. The person in charge of data gathering was a senior emergency medicine resident under the supervision of an emergency medicine specialist.

The duration of procedure was considered from the initiation of reduction until the end of fixating the reduced bone. In addition, time to recovery was considered the time interval between the end of the procedure until complete regain of consciousness and awakening of the patient

***Statistical analysis***

For analyzing data, SPSS 21 statistical software was used. For reporting data, frequency and percentage, or mean ± standard deviation were used. Minimum required sample size for performing the study was determined as 40 patients in each group considering type 1 error of 5%, 95% power, 96% and 67% probability of failure for the 2 groups (12) and the minimum clinically significant difference of 30%. To compare the results between the 2 groups, statistical tests including t-test, chi-square, and Fisher’s exact test were applied. In addition, the rate of absolute risk increase and number needed to harm of treatment failure in case of using dexmedetomidine instead of midazolam was calculated using a medical calculator and reported. Level of significance was considered to be 5%.

## Results:

***Baseline characteristics of the patients***

80 patients with the mean age of 42.08 ± 12.17 (18 - 60) years were randomly allocated to 2 groups of 40 and studied (83.80% male). Table 1 compares the age and sex distribution of the patients in the 2 groups. Mean age in dexmedetomidine group was 40.65 ± 13.25 and it was 43.52 ± 10.95 years in midazolam group (p = 0.294). All the studied patients had a pain severity equal to or greater than 6 in the beginning of reduction. Mean pain severity in dexmedetomidine and midazolam groups before reduction was 8.28 ± 1.13 and 8.18 ± 1.08, respectively (p = 0.688).

***Response to treatment***

Overall, 71 (88.80%) patients experienced 3 points decrease in pain score after receiving the drug. Out of the 9 patients who experienced treatment failure, 8 (88.90%) patients were in dexmedetomidine group and 1 (11.10%) was in the midazolam group (p = 0.029). Table 2 compares mean pain severity at the time of reduction, mean duration of reduction and mean time to recovery between the 2 groups. Mean pain score at the time of reduction was not significantly different between the 2 groups from a clinical point of view; however, dexmedetomidine group had a significantly shorter time to recovery (p < 0.001). None of the patients in either group experienced any special side effects including apnea, nausea and vomiting, or hypotension. Only 3 (7.5%) patients in dexmedetomidine group experience a short episode of bradycardia, which was resolved by slowing the infusion rate from 10 minutes to 15-20 minutes without hemodynamic impairment.

Absolute risk increase rate of treatment failure in case of using dexmedetomidine instead of midazolam was 17.50% (95%CI: 4.19 – 30.81) and number needed to harm was 6.00 (95% CI: 3.20 – 23.80).

**Table 1:** Comparison of age and sex distribution of the participants in the 2 studied groups

Variable	**Group n (%)**		P
	Dexmedetomidine/fentanyl	**Midazolam/fentanyl**	
**Sex **			
Male	33 (85.0)	34 (82.5)	0.762
Female	7 (15.0)	6 (17.5)	
**Age **			
18-45	16 (40.0)	9 (22.5)	0.120
45-55	5 (12.5)	11 (27.5)	
55 ≤	19 (47.5)	20 (50.0)	

**Table 2:** Comparing mean pain severity at the time of reduction initiation (15 minutes after sedation), mean procedure duration and mean time to recovery between the 2 studied groups

**Variable **	Groups		**P**
	Dexmedetomidine	**Midazolam**	
**Response to treatment**	32 (80.0)	39 (97.5)	0.029
**Treatment failure**	8 (20.0)	1 (2.5)	
**Pain severity at the time of reduction**	3.47 ± 1.37	2.85 ± 1.05	0.025
**Procedure duration (minutes)**	12.57 ± 1.75	12.60 ± 1.78	0.950
**Time to recovery**	6.60 ± 1.86	12.70 ± 1.70	< 0.001

Pain severity according to Visual analogue scale. Data were presented as mean ± standard deviation or number (%).

## Discussion:

Based on the results of the present study, although the combination of dexmedetomidine and fentanyl had a shorter time to recovery compared to midazolam and fentanyl for induction of sedation and analgesia, the treatment failure rate in case of using dexmedetomidine with 1 µg/kg increased 17.5% and about 1 out of each 6 patients needed a rescue dose. 

After approval of dexmedetomidine as a sedative drug, various studies have been done regarding the effectiveness of this drug and comparing it to other sedative drugs. A comparison between this drug and propofol has indicated the similar sedative effects of both drugs despite the lower effect of dexmedetomidine on the respiratory system and hemodynamics (14, 15).

Senoglu et al. in a clinical trial on 40 patients in need of sedation for non-invasive ventilation, prescribed midazolam for 1 group and dexmedetomidine for the other. Their findings indicated the equal sedative effects of both drugs; however, the group that received dexmedetomidine needed less dose adjustment in comparison to midazolam (16). Dexmedetomidine has been successfully used in providing a conscious sedation state without any respiratory distress and hemodynamic instability throughout fiberoptic bronchoscopy (17).

Zeyneloglu et al. compared dexmedetomidine with midazolam/fentanyl combination in inducing sedation for extracorporeal shock wave lithotripsy (ESWL) and showed that the group receiving dexmedetomidine needed more rescue doses and also more time to recovery (12). However, the findings of the present study were in line with the mentioned one regarding more need for rescue doses; however, they do not agree regarding time to recovery.

In addition, Vazquez et al. compared midazolam and dexmedetomidine for induction of sedation for endoscopy of the upper digestive system and indicated that the group that received dexmedetomidine had a significantly shorter time to recovery and higher satisfaction. In this study, no difference was reported between the 2 drugs regarding side effects and sedative properties (11). The findings of this study was in agreement with the results of the present study, yet as reported in the results section, the patients receiving dexmedetomidine had a significantly higher rate of treatment failure and more need for a rescue dose.

Regarding the side effects of these drugs on respiratory system and hemodynamics there isn’t much of an agreement between the studies. Although the findings of the study by Frolich et al. indicate the superiority of midazolam for having less effect on the mentioned systems (10), Shukry et al. believe that when less effect on the airway and need for faster awakening are priorities for the in-charge physician and the patient, dexmedetomidine seems to be a proper choice (9).

The reason for these differences could be the different ethnic characteristics of the studied patients, different areas in which the drugs were used, different doses of drugs, different drug compounds and…. However, there is not much experience regarding use of dexmedetomidine specially in ED and for induction of sedation and analgesia and to reach a final decision on points such as time to recovery, side effects of the drug on hemodynamics and respiratory system as well as success and failure rates, further studies are required. Performing multi-center studies with a big sample size and considering all the principles of clinical trials might help in this regard.

***Limitations***

Not using double dummy blinding and the probability of the person gathering data being aware of the drugs used can be mentioned as the most important limitations of this study. Additionally, not following patients regarding probable digestive and other side effects during the hours after sedation are among other limitations of the present study.

## Conclusion:

Based on the results of the present study, although the combination of dexmedetomidine and fentanyl had a shorter time to recovery compared to midazolam and fentanyl for induction of sedation and analgesia, the treatment failure rate in case of using dexmedetomidine with 1 µg/kg increased 17.5% and about 1 out of each 6 patients needed a rescue dose. 
